# Metformin Attenuates Cardiac Hypertrophy *Via* the HIF-1α/PPAR-γ Signaling Pathway in High-Fat Diet Rats

**DOI:** 10.3389/fphar.2022.919202

**Published:** 2022-06-27

**Authors:** Yuansheng Liu, Qian Zhang, Lei Yang, Wencong Tian, Yinan Yang, Yuhang Xie, Jing Li, Liang Yang, Yang Gao, Yang Xu, Jie Liu, Yachen Wang, Jie Yan, Guoxun Li, Yanna Shen, Zhi Qi

**Affiliations:** ^1^ Department of Molecular Pharmacology, School of Medicine, Nankai University, Tianjin, China; ^2^ Department of Colorectal Surgery, Tianjin Union Medical Center, Tianjin, China; ^3^ Tianjin Institute of Acute Abdominal Diseases of Integrated Traditional Chinese and Western Medicine, Tianjin Nankai Hospital, Tianjin, China; ^4^ Tianjin Central Hospital of Gynecology Obstetrics, Tianjin, China; ^5^ Xinjiang Production and Construction Corps Hospital, Urumqi, China; ^6^ Tianjin Union Medical Center, Tianjin, China; ^7^ Department of Microbiology, School of Laboratory Medicine, Tianjin Medical University, Tianjin, China; ^8^ Tianjin Key Laboratory of General Surgery in Construction, Tianjin Union Medical Center, Tianjin, China

**Keywords:** coronary artery disease, cardiac hypertrophy, high-fat diet, metformin, HIF-1α, PPAR-γ

## Abstract

Coronary artery disease (CAD) and cardiac hypertrophy (CH) are two main causes of ischemic heart disease. Acute CAD may lead to left ventricular hypertrophy (LVH). Long-term and sustained CH is harmful and can gradually develop into cardiac insufficiency and heart failure. It is known that metformin (Met) can alleviate CH; however, the molecular mechanism is not fully understood. Herein, we used high-fat diet (HFD) rats and H9c2 cells to induce CH and clarify the potential mechanism of Met on CH. We found that Met treatment significantly decreased the cardiomyocyte size, reduced lactate dehydrogenase (LDH) release, and downregulated the expressions of hypertrophy markers ANP, VEGF-A, and GLUT1 either *in vivo* or *in vitro*. Meanwhile, the protein levels of HIF-1α and PPAR-γ were both decreased after Met treatment, and administrations of their agonists, deferoxamine (DFO) or rosiglitazone (Ros), markedly abolished the protective effect of Met on CH. In addition, DFO treatment upregulated the expression of PPAR-γ, whereas Ros treatment did not affect the expression of HIF-1α. In conclusion, Met attenuates CH via the HIF-1α/PPAR-γ signaling pathway.

## Introduction

Coronary artery disease (CAD) is one of the major cardiovascular diseases, which has been found to be the leading cause of death in both developed and developing countries (McCullough, 2007). CAD is an atherosclerotic disease which is inflammatory in nature, manifested by stable angina, unstable angina, myocardial infarction (MI), or sudden cardiac death (Malakar et al., 2019). Cardiac hypertrophy (CH) refers to the cardiac remodeling of the heart in response to various stresses and stimuli, and it is characterized by the increase of cardiomyocyte size and the thickening of ventricular walls (Gupta et al., 2007). CH is an adaptive response of the heart to maintain normal cardiac efficiency and function. However, long-term and persistent CH is harmful and can result in cardiac insufficiency and heart failure in its further development (Nakamura and Sadoshima, 2018). CAD and left ventricular hypertrophy (LVH) are two common causes of ischemic heart disease (IHD), and LVH can further increase morbidity and mortality due to myocardial infarction. Therefore, the latest management of both the acute and chronic phases of CAD places an increased emphasis on controlling the predisposing factors to prevent or reverse LVH (Khalid et al., 2021). Another study reported that in acute anemic CAD, the hemodynamic changes found may contribute to LVH if the anemic state persists chronically (Rymer and Rao, 2018). Therefore, it is of great significance to find an effective therapeutic strategy for CH and elucidate its molecular mechanism.

Metformin (Met) has been the first-line oral anti-hyperglycemic agent for type 2 diabetes mellitus (T2DM) for decades (Sanchez-Rangel and Inzucchi, 2017). Met has a superior safety profile and tolerance in T2DM treatment (Foretz et al., 2014). In addition to its remarkable glucose-lowering effect, Met has extra advantages in other aspects, including cancers, liver diseases, and renal diseases (Bhat et al., 2015; Morales and Morris, 2015; Podhorecka et al., 2017; Lv and Guo, 2020), especially in the cardiovascular system. It is worth noting that Met has a cardiovascular protective effect on diabetic patients independent of its blood glucose–lowering effect (Zilov et al., 2019; Li J. et al., 2020; Mohan et al., 2021). Some studies have shown that Met can activate AMPK and thereby resist CH by reducing O-GlcNAcylation (Gélinas et al., 2018a; Gélinas et al., 2018b), or AMPK activates SIRT2 to decrease aging-related and Ang II–induced CH (Tang et al., 2017). The protective effect of Met on CH has not been fully elucidated.

Hypoxia-inducible factor 1 (HIF-1), a transcription factor, is a key regulator of oxygen homeostasis genes in metazoan species (Semenza, 2014). HIF-1 binds to hypoxia response elements, and thereafter activates the transcription of many genes, including hematopoiesis, angiogenesis, vascular endothelial growth factor, nitric oxide synthases, glycolytic enzymes, glucose transporters, iron metabolism, and so on (O'Rourke et al., 1999). HIF-1 controls both oxygen delivery by regulating angiogenesis and vascular remodeling, and oxygen utilization by regulating glucose metabolism and redox homeostasis (O'Rourke et al., 1999). HIF-1 is a heterodimer that is composed of an O_2_-regulated HIF-1α subunit and a constitutively expressed HIF1-β subunit (Wang et al., 1995). The activation of HIF-1 is mediated predominantly by post-translational processes of the α subunit (Huang et al., 1996). For decades, scientists have explored the role of HIF-1α in cardiovascular protection. For example, HIF-1 has been shown to play a key protective role in the pathophysiology of ischemic heart disease and stress-induced heart failure (Sousa Fialho et al., 2019); other studies have shown that partial HIF-1α deficiency is harmful and may lead to congenital heart defects in humans; Gao et al., 2015 have demonstrated that when cardiomyocytes are exposed to high glucose *in vitro*, HIF-1α is involved in regulating SOX9 expression and thus effectively regulating and protecting cardiomyocyte hypertrophy; and another study has confirmed that HIF-1α can regulate pathological CH and participate in the regulation of cardiac remodeling (Passariello et al., 2015). It is reported that partial HIF-1α deficiency may be associated with congenital heart defects in humans (Lage et al., 2012). Therefore, studies on the protective effect of HIF-1α on cardiovascular and cardiac functions still have conflicting results, and the mechanism through which HIF-1α regulates HFD-induced CH in animals remains unclear, which requires further research to clarify.

Peroxisome proliferator-activated receptor γ (PPAR-γ), an important regulator of adipocyte differentiation and metabolism, is a member of the nuclear receptor superfamily (Lefterova et al., 2014). PPAR-γ is implicated in insulin sensitivity and metabolic syndrome; therefore, studies pay attention to its therapeutic potential in T2DM. A recent study has shown that the PPAR-γ-PI3K/AKT-GLUT4 signaling pathway plays a role in increasing glucose uptake and decreasing insulin resistance in HFD-fed mice and 3T3-L1 adipocytes (Chen et al., 2019). Meanwhile, the role of PPAR-γ has been studied in the field of diabetic cardiomyopathy (DCM). Interestingly, the role of PPAR-γ in DCM is controversial. Yan et al. (2018) demonstrated that PPAR-γ activation in the heart of obese diabetic mice can decrease heart fibrosis. On the other hand, rosiglitazone (Ros), an activator of PPAR-γ, promoted hypertrophy in primary neonatal rat cardiomyocytes (NRCM) (Pharaon et al., 2017). Therefore, it is essential to further clarify the role of PPAR-γ in the progression of CH.

The interaction between Met and the HIF-1α or PPAR-γ signaling pathway during CH has not been reported yet. In this study, we intend to examine the protective effect of Met on CH and clarify whether the mechanism is related to the HIF-1α or PPAR-γ signaling pathway.

## Materials and Methods

### Animals

Male Sprague–Dawley (SD) rats weighing 180–200 g were purchased from the Military Academy of the Medical Science Laboratory Animal Center (Beijing, China). Room temperature was maintained at 22–24°C and the rats were housed on a 12-h light-dark cycle. Eighteen rats were randomly divided into two groups, namely, the normal diet (Con, *n* = 6) group and the high-fat diet (HFD) (initial HFD, *n* = 12) group. Rats in the Con group received normal diet feeding and HFD rats received high-fat diet (D12492, New Brunswick, NJ) feeding. After feeding for 28 weeks, the rats in the initial HFD group were further randomized into the following two groups: rats treated with metformin (30 mg/kg/day [Jaikumkao et al., 2018]) (HFD + Met, *n* = 6) or without metformin (HFD, *n* = 6) for another 6 weeks. All rats were sacrificed on the 34th week, and the rats’ hearts, blood, and adipose tissues were collected for further assessments. All animal experiments were performed strictly following the guidelines on laboratory animals of Nankai University, and were approved by the Institute Research Ethics Committee at the Nankai University (Permit number: 10011).

### Echocardiography

Left ventricle (LV) geometry and functions were evaluated in the rats by two-dimensional (2D)–guided M-mode echocardiography (VisualSonics Vevo 2100, 30 MHz linear signal transducer, Visual Sonics). Averaged M-mode measurements from parasternal long-axis images were recorded. Interventricular septal thickness (IVS), left ventricular posterior wall (LVPW) dimensions, left ventricular internal dimensions (LVID), and the left atrial volume index (LV Vol) were taken in diastole and systole.

### Quantitative Real-Time Polymerase Chain Reaction

The protocol for qPCR was described previously (Feng et al., 2022). Briefly, the total RNA of rats’ heart samples was isolated using Trizol reagent (Takara Bio, Japan.). For cDNA synthesis, 1.0 µg RNA was used and the reactions were carried out using the reverse transcription system (Promega, China). The qPCR was performed using SYBR Green Master Mix (Promega, China) in a Bio-Rad IQ5 detection system, and the cycle threshold (CT) values were automatically determined in triplicates and averaged.

### H-E Staining

The left ventricles were fixed with 4% paraformaldehyde for 24 h at room temperature. The tissues were dehydrated and embedded in paraffin. Then, 5-mm sections were cut from the paraffin blocks and stained with hematoxylin and eosin (H-E) for histopathological examination.

### Cell Culture

The fetal rat cardiomyocyte-derived cell line (H9c2) was purchased from Shanghai Institutes for Biological Sciences (Shanghai, China). H9c2 cells were cultured in Dulbecco’s minimal essential medium (DMEM, pH 7.2) supplemented with 10% heat-inactivated fetal bovine serum (FBS), 100 U/mL of penicillin, and 100 μg/ml of streptomycin in 95% air and 5% CO_2_ at 37°C. The culture medium was changed every 2 days and the cells were subcultured once they attained 80% confluence.

H9c2 cells were divided into five groups, namely, the control (Con) group, palmitate treatment (Pa, 75 μM for 3 h [Yamamoto et al., 2020], Sigma) group, deferoxamine treatment (DFO, 150 μM for 18 h [Hopfner et al., 2020], Sigma) group, palmitate plus metformin treatment (Pa + Met, Met, 1 mM for 18 h [Zhang et al., 2018], Sigma) group, and palmitate plus metformin and deferoxamine combination treatment (Pa + Met + DFO) group.

In another *in vitro* experiment, H9c2 cells were divided into five groups, namely, the Con group, Pa group, rosiglitazone treatment (Ros, 100 μM for 48 h (Pharaon et al., 2017), MCE) group, Pa + Met group, and palmitate plus metformin and rosiglitazone combination treatment (Pa + Met + Ros) group.

### Western Blot Analysis

The protocol for Western blot analysis was described previously (Li G. et al., 2020). In brief, left ventricle samples and cell samples were lysed in ice-cold RIPA lysis buffer and centrifuged. The concentration of total protein was measured by using a BCA protein assay kit (Thermo, United States). The protein sample (20–50 µg) was separated by 10% SDS-PAGE and then transferred onto a polyvinylidene fluoride membrane (Merck KGaA, Darmstadt, Germany). After blocking with 5% BSA for 2 h at room temperature, the membranes were incubated overnight at 4°C with the following primary antibodies: HIF-1α (Cell signaling technology, United States), PPAR-γ (Immunoway, United States), GLUT1 (Abcam, United Kingdom), VEGF-A (Abcam, United Kingdom), ANP, and *ß*-actin (Santa Cruz, CA). The results were expressed as fold changes normalized to *ß*-actin. Anti-rabbit and anti-mouse IgG with a peroxidase-conjugated antibody were used as secondary antibodies (Promega, Shanghai). Band densities were quantified using ImageJ software.

### Immunofluorescence Staining

After drug treatment, H9c2 cells were seeded on coverslips and permeabilized with 0.5% Triton X-100 for 15 min at room temperature, blocked with 5% goat serum for 1 h at room temperature and incubated with appropriate primary antibodies, ANP or α-actin (Thermo Fisher Scientific, United States), overnight at 4°C. The samples were washed three times with TBST followed by incubation with Alexa Fluor 488– or Alexa Fluor 594–conjugated secondary antibodies (1:200, Cell signaling technology, United States) for 1 h at room temperature. Nuclei were stained with DAPI (Sigma, United States) for 2 min at room temperature. Images were acquired with an FV1000 confocal microscope (Olympus, Japan) and analyzed with ImageJ software.

### TUNEL Staining

Terminal deoxynucleotidyl transferase-mediated nick end labeling (TUNEL) staining in H9c2 cells was performed according to the protocol of the TUNEL staining kit (Roche, United States) to determine the apoptosis of cardiomyocytes as previously described (Tian et al., 2020).

### LDH and CK-MB Release

Serum lactate dehydrogenase (LDH) and creatine kinase isoenzyme (CK-MB) activities were measured by using an automated clinical chemistry analyzer (DRI-CHEM NX500, Fujifilm, Japan). The LDH level in the cell culture supernatant was determined using an LDH assay kit (Nanjing Jiancheng, China) with a microplate reader (Thermo, China).

### Statistical Analysis

Results are expressed as mean ± SEM. One-way ANOVA followed by an LSD test was performed in the experiment (SPSS ver. 17). A value of *p* < 0.05 was accepted as statistically significant.

## Results

### Met Treatment Decreased Body Weight, Fat Accumulation, and Triglyceride Level in HFD Rats

**FIGURE 1:**
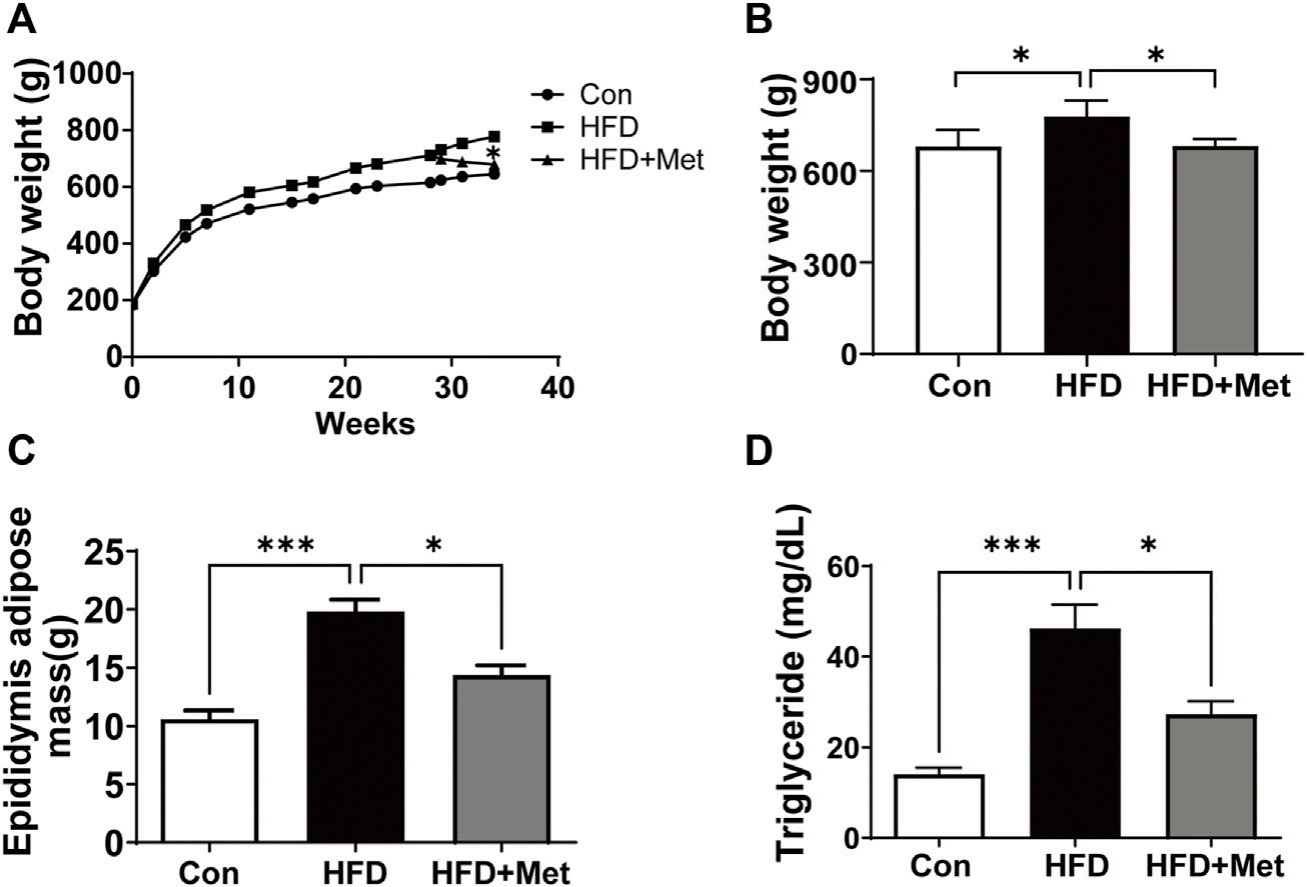
Met treatment decreased the body weight, fat accumulation, and triglyceride level in HFD rats. **(A)** Growth curves. **(B)** Body weight in the 34th week for each group. **(C)** Epididymis adipose mass in the 34th week for each group. **(D)** Serum triglyceride was measured for each group. Graphs represent mean ± SEM (*n* = 6). ^*^ *p* < 0.05, ^***^ *p* < 0.001.

Figure 1A shows the growth curve for three different groups. We found that Met treatment resulted in a significant weight loss in the 34th week in HFD rats. The body weight in the HFD group is 778.2 ± 21.5 g higher than those in the Con group (679.7 ± 31.6 g) in the 34th week, whereas Met treatment significantly reduced the rats’ body weight (681.3 ± 13.3 g in the Met group *vs*. 778.2 ± 21.5 g in the HFD group) (Figure 1B). Similar to the results of body weight, HFD rats showed higher epididymis adipose mass and triglyceride levels in comparison with Con rats. In contrast, Met treatment reduced epididymis adipose mass and serum triglyceride levels when compared with the HFD rats (Figures 1C,D).

### Met Treatment Attenuated HFD-Induced CH

**FIGURE 2:**
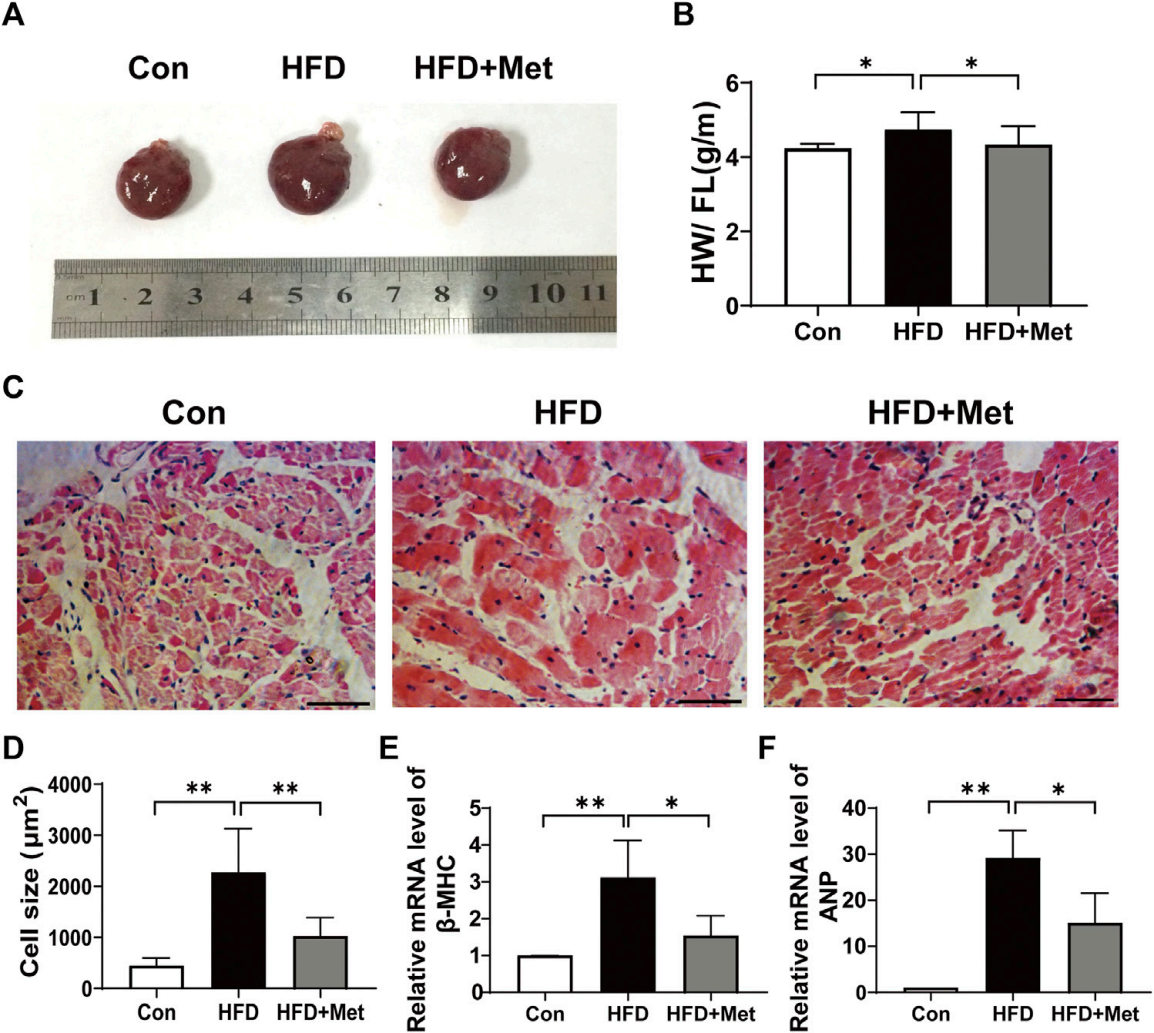
Met treatment attenuated HFD-induced CH. **(A)** Heart morphology. **(B)** Heart weight/femur length. **(C)** HE staining of heart tissue (scale bar: 50 µm). **(D)** Quantification of cardiomyocyte size. **(E,F)** mRNA expression levels of *ß*-MHC and ANP. Graphs represent mean ± SEM (*n* = 6). ^*^ *p* < 0.05, ^**^ *p* < 0.01.

All rats were sacrificed in the 34th week. The rats’ hearts were isolated and heart sizes were measured (Figure 2A). We found that the value of the heart weight to femur length (HW/FL) in the HFD group was significantly higher than that in the Con group, indicating that HFD feeding caused CH. In contrast, HW/FL was markedly decreased in the HFD + Met group (Figure 2B). Consistent with the aforementioned results, H-E staining also showed that the size of cardiomyocytes in the HFD group was larger than that in the Con group, but after the treatment with Met, the cell size significantly became smaller in comparison with the HFD group (Figures 2C,D). Furthermore, the mRNA levels of two markers for CH, namely, *ß*-MHC and ANP, were examined and quantified. As shown in Figures 2E,F, the mRNA levels of *ß*-MHC and ANP were all elevated in the HFD group in comparison with the Con group, whereas their levels were significantly alleviated in the HFD + Met group.

### Met Attenuated HFD-Induced LV Dysfunction

**FIGURE 3:**
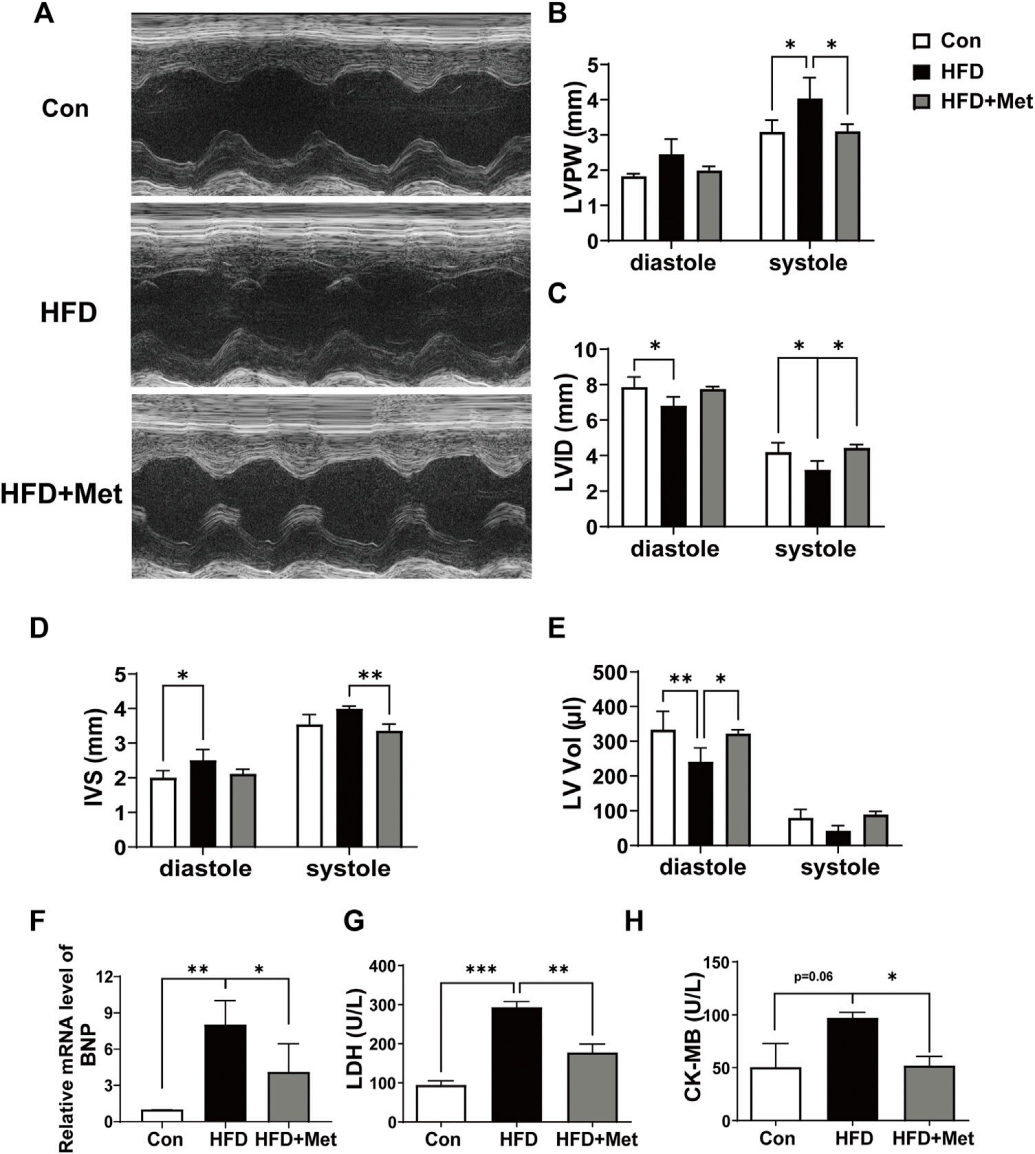
Met attenuated HFD-induced LV dysfunction. **(A)** Representative echocardiography. **(B–E)** Assessment of the left ventricular posterior wall, left ventricular internal dimensions, interventricular septal, and left atrial volume index. **(F)** mRNA expression levels of ANP. **(G,H)** Serum LDH and CK-MB were measured for each group. Graphs represent mean ± SEM (*n* = 6). ^*^ *p* < 0.05, ^**^ *p* < 0.01, ^***^ *p* < 0.001.

Echocardiography was performed in the 34th week to observe CH and assess myocardial function (Figure 3A). HFD rats showed significant incrassation of the ventricular wall and the LVPW, and Met treatment markedly decreased the LVPW when compared with the HFD group (Figures 3A,B). HFD feeding decreased the levels of LVID and LV Vol, and increased the IVS level in comparison with normal diet feeding, while these undesirable changes were notably improved in the HFD + Met group (Figures 3C–E). Meanwhile, Met treatment significantly decreased HFD-induced upregulation of the BNP mRNA level and inhibited the release of LDH and CK-MB in serum (Figures 3F–H). These findings indicated that Met could prevent HFD-induced cardiac LV dysfunction.

### Met Inhibited HIF-1α and PPAR-γ Signaling Pathway in HFD Rats

**FIGURE 4:**
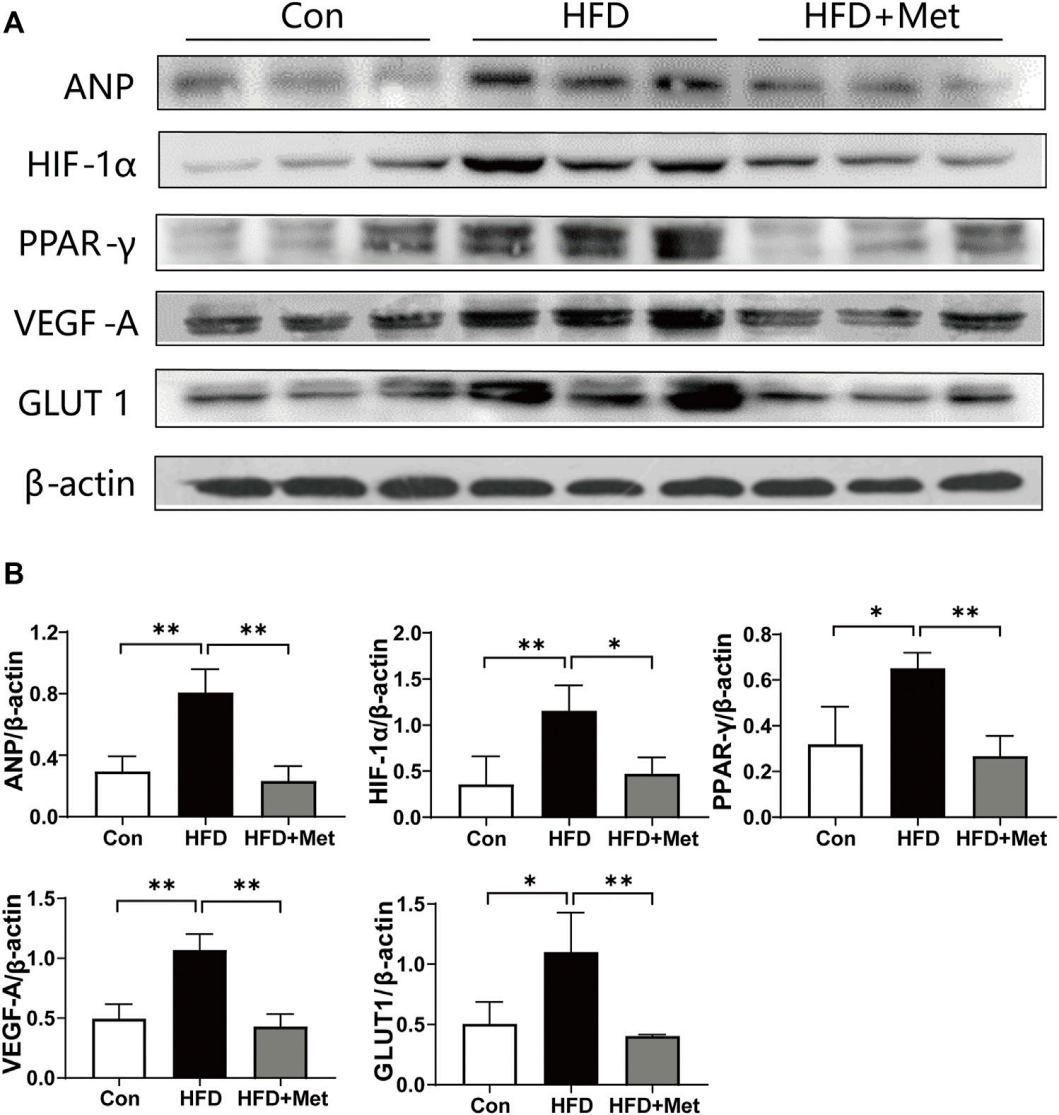
Met inhibited HIF-1α and PPAR-γ signaling pathways in HFD rats. **(A,B)** Western blot analysis of ANP, HIF-1α, PPAR-γ, VEGF-A, and GLUT1 in heart tissue. Graphs represent mean ± SEM (*n* = 6). ^*^ *p* < 0.05, ^**^ *p* < 0.01.

First, we examined the expression of ANP, a recognized marker for CH. We found that Met treatment obviously downregulated the expression of ANP, indicating that Met can alleviate obesity-induced CH. To investigate how Met attenuated CH in HFD rats, we determined the expressions of key molecules related to HIF-1α and the PPAR-γ signaling pathway. We found that the expressions of HIF-1α, PPAR-γ, VEGF-A, and GLUT1 were all upregulated after HFD feeding, whereas Met treatment reversed these upregulations (Figure 4).

### Activation of HIF-1α Abolished the Protective Effect of Met on CH in Rat Cardiomyocytes

**FIGURE 5:**
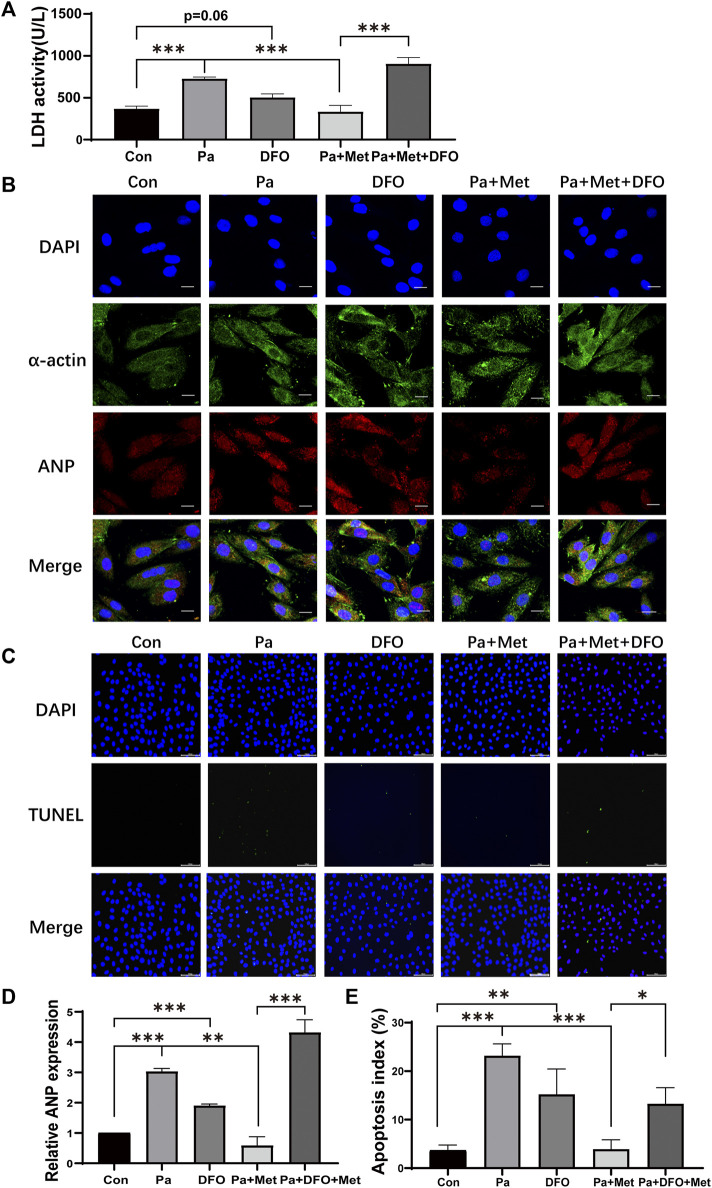
Activation of HIF-1α abolished the protective effect of Met on CH in rat cardiomyocytes. **(A)** LDH activity assessment. **(B)** H9c2 cells were subjected to an immunofluorescence assay with anti-ANP and anti-α-actin antibodies (scale bar: 20 µm). **(C)** Detection of apoptotic cells by TUNEL staining (scale bar: 100 µm). **(D)** Quantitative data of ANP fluorescence intensity analyzed by Image-Pro Plus 6.0 software. **(E)** Quantification of apoptotic cells. Graphs represent mean ± SEM (*n* = 3). ^*^ *p* < 0.05, ^**^ *p* < 0.01, ^***^ *p* < 0.001.

Palmitate (Pa) was used to mimic HFD-feeding *in vitro* experiments. DFO, an agonist for HIF-1α, was added to the culture medium to increase the activity of HIF-1α. Consistent with the results *in vivo*, Pa increased the release of LDH, whereas Met treatment significantly decreased LDH release in the culture supernatant. Interestingly, DFO treatment significantly abolished the weakening effect of Met on LDH release (Figure 5A). Next, we performed an immunofluorescence test for α-actin to label the cytoskeleton and ANP to reflect the degree of cardiomyocyte hypertrophy. As shown in Figures 5B,D, the cells in the Pa group and the DFO group revealed significant hypertrophy, which appealed in the size of the cell and ANP fluorescence intensity, in comparison with the Con group, whereas the cells in the Pa + Met group maintained normal cell morphology. DFO treatment (Pa + Met + DFO) obviously reversed the protective effect of Met on hypertrophy (Figures 5B,D). To examine the CH-induced apoptosis, TUNEL staining was performed in H9c2 cells. Few TUNEL-positive cells were observed in the Con group. In contrast, numerous TUNEL-positive cells were found in the Pa group. Met treatment significantly reduced apoptotic cells in comparison with the Pa group and DFO treatment abolished the protective effect of Met on cardiomyocytes (Figures 5C,E).

### Activation of PPAR-γ Abolished the Protective Effect of Met on CH in Rat Cardiomyocytes

**FIGURE 6:**
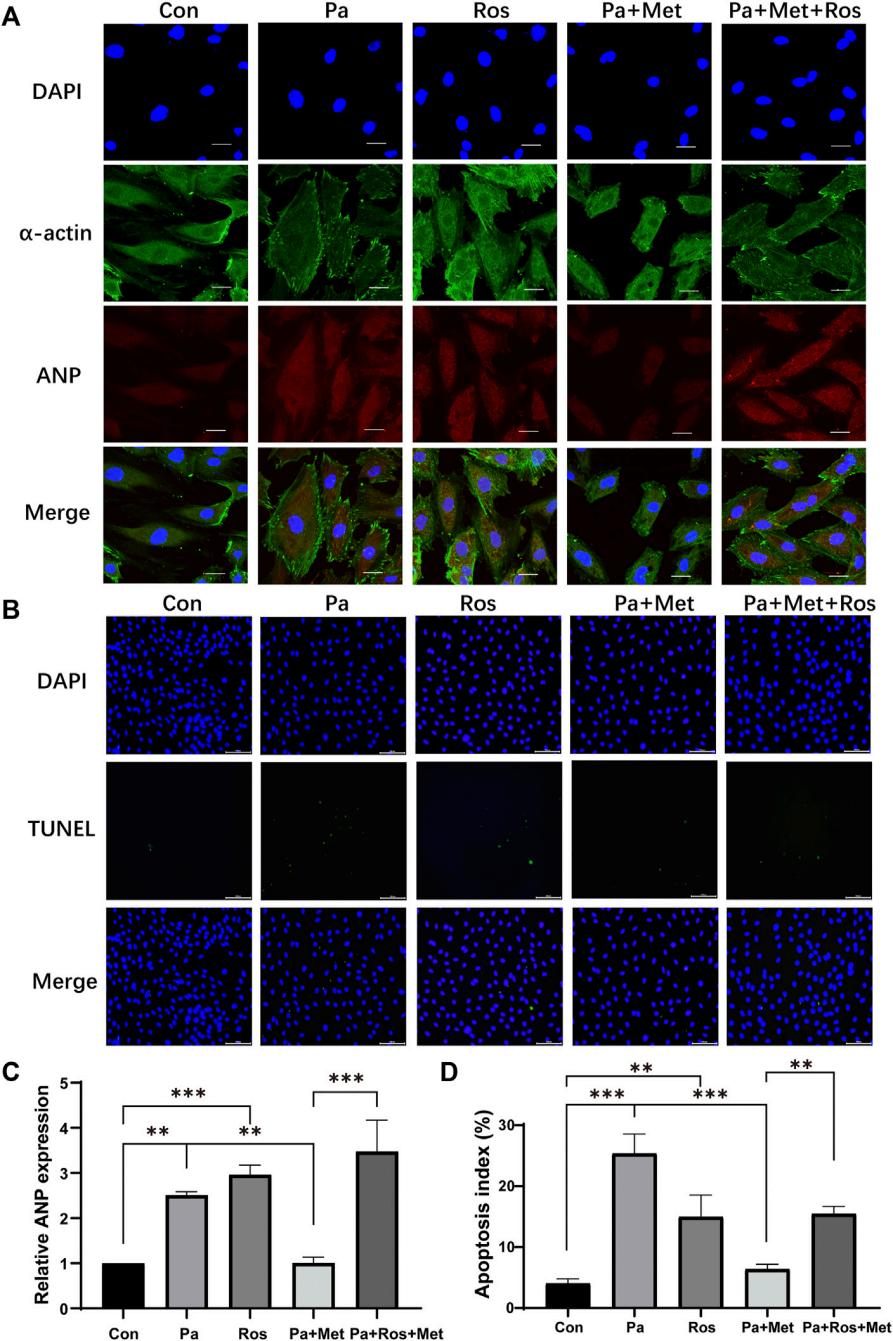
Activation of PPAR-γ abolished the protective effect of Met on CH in rat cardiomyocytes. **(A)** H9c2 cells were subjected to an immunofluorescence assay with anti-ANP and anti-α-actin antibodies (scale bar: 20 µm). **(B)** Detection of apoptotic cells by TUNEL staining (scale bar: 100 µm). **(C)** Quantitative data of ANP fluorescence intensity analyzed by Image-Pro Plus 6.0 software. **(D)** Quantification of apoptotic cells. Graphs represent mean ± SEM (*n* = 3). ^**^ *p* < 0.01, ^***^ *p* < 0.001.

To further clarify whether the protective effect of Met on CH is related to the PPAR-γ signaling pathway, an immunofluorescence test and TUNEL staining were performed *in vitro*. Rosiglitazone (Ros), an activator of PPAR-γ, was used to confirm the role of PPAR-γ in CH. Cells in the Pa or Ros group showed significant hypertrophy and apoptosis in comparison with those in the Con group (Figures 6A–D). Met treatment alleviated hypertrophy and decreased the number of TUNEL-positive cells when compared with the Pa group, whereas the combination of Met and Ros abolished the protective effect of Met in H9c2 cells (Figures 6A–D).

### Met Attenuated CH Through the HIF-1α/PPAR-γ Signaling Pathway

To investigate the role of the HIF-1α/PPAR-γ signaling pathway in Met reducing CH, we upregulated the expression of HIF-1α or PPAR-γ by using their agonists, namely, DFO and Ros in H9c2 cells, respectively.

**FIGURE 7:**
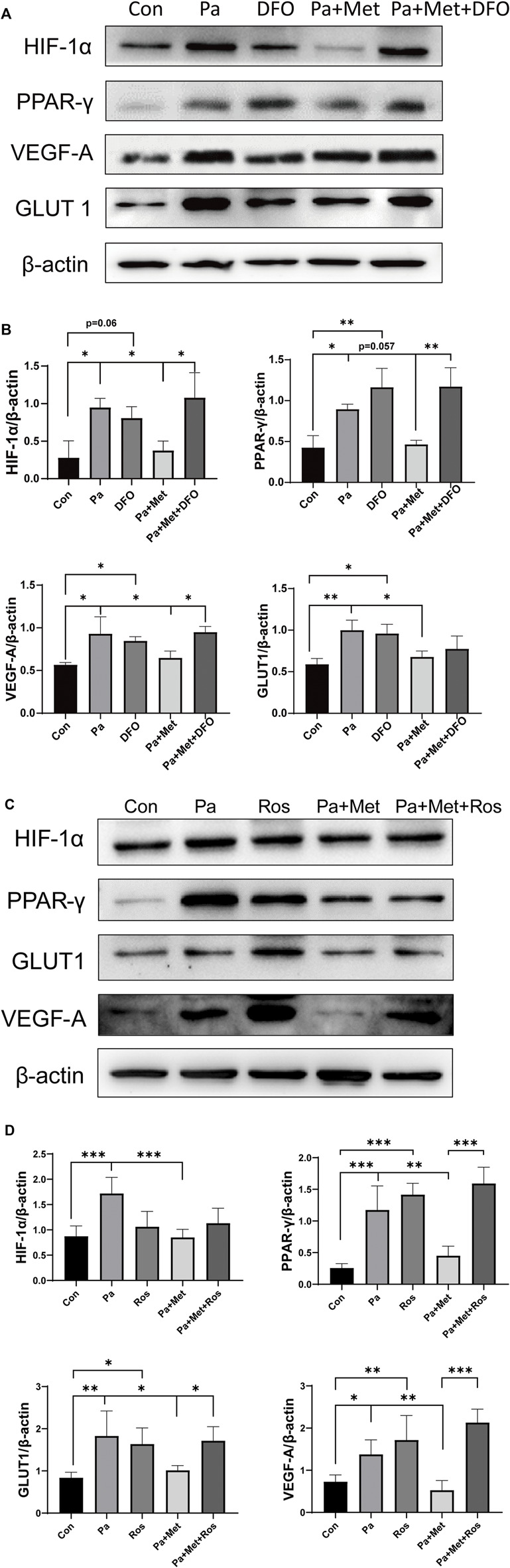
Met attenuated CH through the HIF-1α/PPAR-γ signaling pathway. **(A,B)** Western blot analysis of HIF-1α, PPAR-γ, VEGF-A, and GLUT1 in different H9c2 groups. **(C,D)** Western blot analysis of ANP, HIF-1α, PPAR-γ, VEGF-A, and GLUT1 in different H9c2 groups. Graphs represent mean ± SEM (*n* = 3). ^*^ *p* < 0.05, ^**^ *p* < 0.01.

Pa treatment significantly upregulated the expressions of HIF-1α, PPAR-γ, VEGF-A, and GLUT1 in H9c2 cells. Lower expressions of HIF-1α, PPAR-γ, VEGF-A, and GLUT1 were found in the Pa + Met group in comparison with the Pa or DFO group. In contrast, downregulations of these genes were markedly increased in the Pa + Met + DFO group when compared with the Pa + Met group. (Figures 7A,B).

Interestingly, treatment with Ros, an agonist of PPAR-γ, significantly upregulated the expressions of PPAR-γ, VEGF-A, and GLUT1 in comparison with the Con group, but did not affect the expression of HIF-1α in H9c2 cells. We found that the expressions of HIF-1α, PPAR-γ, VEGF-A, and GLUT1 were downregulated after treatment with Met alone. The combination of Met and Ros significantly increased the expressions of PPAR-γ, VEGF-A, and GLUT1 in comparison with the Pa + Met group, whereas the combination treatment did not rescue the expression of HIF-1α (Figures 7C,D).

### Schematic Model of This Study

Taken together, we found a new signaling pathway for the protective effect of Met on CH. Met attenuates CH via the downregulation of the HIF-1α/PPAR-γ signaling pathway, and then reduces angiogenesis and glycolysis to protect the heart. Therefore, the HIF-1α/PPAR-γ signaling pathway can be used as a potential cardioprotective molecular target in CH.

## Discussion

Met has a significant anti-diabetic effect on T2DM patients with a low price. Therefore, it is used as the first-line oral drug for the treatment of T2DM. Met exerts therapeutic effects against various diseases. Our group found that Met protected the heart against ischemia–reperfusion injury through the activation of the AMPK/antioxidant enzyme signaling pathway (Wang et al., 2017). We also verified that Met inhibited the growth of pancreatic cancer *via* the downregulation of VEGF-B (Zhu et al., 2016). However, the mechanism of Met on CH is not fully understood. In this study, we used HFD rats and H9c2 cells to investigate the effect of Met on CH both *in vivo* and *in vitro*.

Accumulating pieces of evidence showed that a HFD could result in various forms of cardiac abnormalities, including cardiac inflammation, CH, and fibrosis (Sorop et al., 2020; Wali et al., 2020; Zhang and Cai, 2020; Oduro et al., 2022); therefore, an HFD rat is an appropriate animal model for studying CH. We found that HFD rats revealed hallmarks of CH, including increased volume of cardiomyocyte size, upregulated expressions of *ß*-MHC and ANP (Figure 2), and LV dysfunction (Figure 3). It is reported that the administration of Met could prevent weight gain not only in animals but also in humans, especially in obesity-related T2DM patients (Yerevanian and Soukas, 2019; Coll et al., 2020; Chen et al., 2021). In this study, as expected, Met treatment significantly reduced body weight and fat accumulation in comparison with HFD rats (Figure 1). In addition, our results showed that Met treatment significantly reduced CH (Figure 2, Figures 3A,B,F), improved LV functions (Figures 3A–E), and attenuated injury of cardiomyocytes (Figures 3G,H) in HFD rats. Meanwhile, reduced expressions of ANP and decreased TUNEL-positive cells were also observed after Met treatment in H9c2 cells (Figures 5B,C, Figures 6A,B). These results indicated that Met markedly attenuated CH-induced LV dysfunction and cardiac injury either *in vivo* or *in vitro*.

Krishnan et al. (2009)reported that HIF-1α and PPAR-γ were jointly upregulated in hypertrophic cardiomyopathy and cooperated to mediate key changes in cardiac metabolism. Under pathologic stress, HIF-1α activated PPAR-γ, which subsequently resulted in changes in energy metabolism and cardiac hypertrophy (Krishnan et al., 2009). Boutoual et al. (2018)revealed that the HIF–PPARγ–UCP2–AMPK axis played an important role in the metabolic reprogramming of the MTO1- and GTPBP3-defective cells, and these defects caused infantile hypertrophic cardiomyopathy with lactic acidosis. Interestingly, in breast cancer, HIF-1α is activated by PPAR-γ to induct autophagy, and HIF-1α knockout blocks PPAR-γ activation–induced autophagosome formation (Zhou et al., 2009). Therefore, we intend to examine the relationship between HIF-1α and PPAR-γ in this study.

Several studies reported that chronic increase of VEGF-A in the heart led to increased cardiac angiogenesis and development of CH; therefore, VEGF-A has become a recognized marker for CH (Tirziu et al., 2007; Marneros, 2018; Kivelä et al., 2019). In some heart disease models, such as congestive heart failure and valvular heart disease, VEGF-A expression showed a positive correlation with the expression of HIF-1α (Lee et al., 2019) and studies have proved that VEGF-A is one of the downstream targets of HIF-1α indeed (MacDonald et al., 2013; Wen et al., 2019).

Glucose transporters (GLUT) are essential for the heart to sustain its function. It is markedly affected in cardiac diseases such as CH, DCM, and heart failure (Heitmeier et al., 2018). Pathological hypertrophy and its frequent outcome of heart failure are associated with a metabolic remodeling where glucose becomes the main and constant ATP-generating substrate (Lopaschuk and Ussher, 2016; Peterzan et al., 2017). This alteration is due to a complex reorganization of the gene expression profile that resembles the fetal pattern, in which GLUT1 expression is increased and GLUT4 is downregulated (Bertrand et al., 2020). A study also proved that GLUT1 was the downstream gene of HIF-1α in T2DM renal disease (Huang et al., 2020). The increase in GLUT1 expression is conducive to glucose intake, which is an adaptive response to cardiac hypertrophy; hence, GLUT1 was already considered to be another marker for CH (Bertrand et al., 2020). Interestingly, a study found that PPAR-γ suppression reduced insulin-stimulated glucose uptake in adipocytes, which was mediated by both GLUT4 and GLUT1 (Liao et al., 2007). GLUT transgenic and knockout mice have provided valuable insight into the role of facilitative GLUTs in cardiovascular and metabolic diseases. Heitmeier et al. (2018) reported that compensatory metabolic adaptation in response to chronic GLUT blockade could evade deleterious changes in the failing heart. In a word, GLUT played an important role in both HIF-1α and PPAR-γ signaling pathways.

Although numerous studies have reported the cardioprotective effect of Met, to our knowledge, this novel study is the first one to investigate the cardioprotective effect of Met against CH through the HIF-1α/PPAR-γ signaling pathway. We first examined the protein expressions of HIF-1α, PPAR-γ, GLUT1, and VEGF-A in the heart tissue of HFD rats. The four aforementioned genes showed the same tendency, that is. they were all increased in HFD rats and reduced after Met treatment (Figure 4). To further clarify the role of the HIF-1α/PPAR-γ signaling pathway in CH, we then performed *in vitro* experiments in H9c2 cells. According to the results of the LDH release test and immunofluorescence, we found that the combination of DFO and Met significantly abolished the protective effect of Met on cardiac injury (Figure 5A), CH (Figure 5B), and apoptosis (Figure 5C), indicating that Met exerted its protective effect on CH in an HIF-1α–dependent manner. Meanwhile, Ros, the PPAR-γ agonist treatment, markedly reversed the alleviating effect of Met on CH (Figure 6A) and apoptosis (Figure 6B) in H9c2 cells, indicating that Met attenuated CH depending on the expression of PPAR-γ.

To further investigate the relationship between HIF-1α, PPAR-γ, GLUT1, and VEGF-A, activation of HIF-1α or PPAR-γ was performed by DFO or Ros treatment in H9c2 cells, respectively. DFO or Ros treatment significantly upregulated the expression of HIF-1α or PPAR-γ, VEGF-A, and GLUT1. Similar to the results in HFD rats, Met treatment significantly downregulated the expressions of HIF-1α, PPAR-γ, GLUT1, and VEGF-A. Interestingly, either DFO or Ros treatment with Met obviously increased the expressions of GLUT1 and VEGF-A in comparison with treatment with Met alone (Pa + Met), indicating that GLUT1 and VEGF-A are the down-stream genes of HIF-1α and PPAR-γ. Importantly, DFO treatment up-regulated the expression of PPAR-γ, whereas Ros treatment did not affect the expression of HIF-1α, indicating that HIF-1α might be the up-stream of PPAR-γ (Figures 6, 7).

**FIGURE 8:**
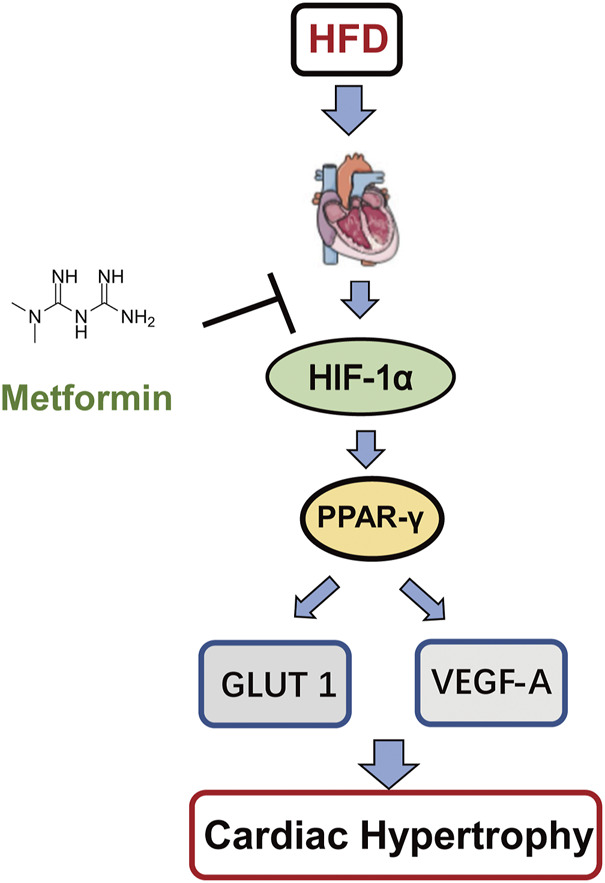
Schematic model of this study. Met protects CH through the downregulation of the HIF-1α/PPAR-γ signaling pathway.

To our knowledge, this is the first time that the relationship between Met treatment and the HIF-1α/PPAR-γ signaling pathway during CH is explored. In conclusion, Met treatment attenuated CH, improved LV function, and reduced apoptosis of cardiomyocytes not only in HFD rats but also in Pa-treated H9c2 cells. This cardioprotective effect of Met depended on the HIF-1α/PPAR-γ signaling pathway (Figure 8). Therefore, Met can be used as a potential cardioprotective adjuvant in CH therapy and the inhibition of the HIF-1α/PPAR-γ signaling pathway will be a promising modality for clinical CH therapy.

## Data Availability

The raw data supporting the conclusion of this article will be made available by the authors, without undue reservation.

## References

[B1] Bertrand L. Auquier J. Renguet E. Angé M. Cumps J. Horman S. (2020). Glucose Transporters in Cardiovascular System in Health and Disease. Pflugers Arch. 472 (9), 1385–1399. 10.1007/s00424-020-02444-8 32809061

[B2] Bhat A. Sebastiani G. Bhat M. (2015). Systematic Review: Preventive and Therapeutic Applications of Metformin in Liver Disease. World J. Hepatol. 7 (12), 1652–1659. 10.4254/wjh.v7.i12.1652 26140084 PMC4483546

[B3] Boutoual R. Meseguer S. Villarroya M. Martín-Hernández E. Errami M. Martín M. A. (2018). Defects in the Mitochondrial-tRNA Modification Enzymes MTO1 and GTPBP3 Promote Different Metabolic Reprogramming through a HIF-Pparγ-UCP2-AMPK axis. Sci. Rep. 8 (1), 1163. 10.1038/s41598-018-19587-5 29348686 PMC5773609

[B4] Chen T. Zhang Y. Liu Y. Zhu D. Yu J. Li G. (2019). MiR-27a Promotes Insulin Resistance and Mediates Glucose Metabolism by Targeting PPAR-γ-Mediated PI3K/AKT Signaling. Aging (Albany NY) 11 (18), 7510–7524. 10.18632/aging.102263 31562809 PMC6781997

[B5] Chen X. He S. Wang D. (2021). Effects of Metformin on Body Weight in Polycystic Ovary Syndrome Patients: Model-Based Meta-Analysis. Expert Rev. Clin. Pharmacol. 14 (1), 121–130. 10.1080/17512433.2021.1863788 33306918

[B6] Coll A. P. Chen M. Taskar P. Rimmington D. Patel S. Tadross J. A. (2020). GDF15 Mediates the Effects of Metformin on Body Weight and Energy Balance. Nature 578 (7795), 444–448. 10.1038/s41586-019-1911-y 31875646 PMC7234839

[B7] Feng L. Li G. An J. Liu C. Zhu X. Xu Y. (2022). Exercise Training Protects against Heart Failure via Expansion of Myeloid-Derived Suppressor Cells through Regulating IL-10/STAT3/S100A9 Pathway. Circ. Heart Fail 15 (3), e008550. 10.1161/circheartfailure.121.008550 34911348

[B8] Foretz M. Guigas B. Bertrand L. Pollak M. Viollet B. (2014). Metformin: from Mechanisms of Action to Therapies. Cell. Metab. 20 (6), 953–966. 10.1016/j.cmet.2014.09.018 25456737

[B9] Gao Q. Guan L. Hu S. Yao Y. Ren X. Zhang Z. (2015). Study on the Mechanism of HIF1a-SOX9 in Glucose-Induced Cardiomyocyte Hypertrophy. Biomed. Pharmacother. 74, 57–62. 10.1016/j.biopha.2015.07.009 26349963

[B10] Gélinas R. Dontaine J. Horman S. Beauloye C. Bultot L. Bertrand L. (2018a). AMP-activated Protein Kinase and O-GlcNAcylation, Two Partners Tightly Connected to Regulate Key Cellular Processes. Front. Endocrinol. (Lausanne) 9, 519. 10.3389/fendo.2018.00519 30271380 PMC6146136

[B11] Gélinas R. Mailleux F. Dontaine J. Bultot L. Demeulder B. Ginion A. (2018b). AMPK Activation Counteracts Cardiac Hypertrophy by Reducing O-GlcNAcylation. Nat. Commun. 9 (1), 374. 10.1038/s41467-017-02795-4 29371602 PMC5785516

[B12] Gupta S. Das B. Sen S. (2007). Cardiac Hypertrophy: Mechanisms and Therapeutic Opportunities. Antioxid. Redox Signal 9 (6), 623–652. 10.1089/ars.2007.1474 17511580

[B13] Heitmeier M. R. Payne M. A. Weinheimer C. Kovacs A. Hresko R. C. Jay P. Y. (2018). Metabolic and Cardiac Adaptation to Chronic Pharmacologic Blockade of Facilitative Glucose Transport in Murine Dilated Cardiomyopathy and Myocardial Ischemia. Sci. Rep. 8 (1), 6475. 10.1038/s41598-018-24867-1 29691457 PMC5915485

[B14] Hopfner U. Maan Z. N. Hu M. S. Aitzetmüller M. M. Zaussinger M. Kirsch M. (2020). Deferoxamine Enhances the Regenerative Potential of Diabetic Adipose Derived Stem Cells. J. Plast. Reconstr. Aesthet. Surg. 73 (9), 1738–1746. 10.1016/j.bjps.2020.02.045 32418841

[B15] Huang L. E. Arany Z. Livingston D. M. Bunn H. F. (1996). Activation of Hypoxia-Inducible Transcription Factor Depends Primarily upon Redox-Sensitive Stabilization of its Alpha Subunit. J. Biol. Chem. 271 (50), 32253–32259. 10.1074/jbc.271.50.32253 8943284

[B16] Huang Y. Jin L. Yu H. Jiang G. Tam C. H. T. Jiang S. (2020). SNPs in PRKCA-Hif1a-GLUT1 Are Associated with Diabetic Kidney Disease in a Chinese Han Population with Type 2 Diabetes. Eur. J. Clin. Invest. 50 (9), e13264. 10.1111/eci.13264 32394523

[B17] Jaikumkao K. Pongchaidecha A. Chueakula N. Thongnak L. Wanchai K. Chatsudthipong V. (2018). Renal Outcomes with Sodium Glucose Cotransporter 2 (SGLT2) Inhibitor, Dapagliflozin, in Obese Insulin-Resistant Model. Biochim. Biophys. Acta Mol. Basis Dis. 1864 (6 Pt A), 2021–2033. 10.1016/j.bbadis.2018.03.017 29572114

[B18] Khalid K. Padda J. Ismail D. Abdullah M. Gupta D. Pradeep R. (2021). Correlation of Coronary Artery Disease and Left Ventricular Hypertrophy. Cureus 13 (8), e17550. 10.7759/cureus.17550 34646607 PMC8479854

[B19] Kivelä R. Hemanthakumar K. A. Vaparanta K. Robciuc M. Izumiya Y. Kidoya H. (2019). Endothelial Cells Regulate Physiological Cardiomyocyte Growth *via* VEGFR2-Mediated Paracrine Signaling. Circulation 139 (22), 2570–2584. 10.1161/circulationaha.118.036099 30922063 PMC6553980

[B20] Krishnan J. Suter M. Windak R. Krebs T. Felley A. Montessuit C. (2009). Activation of a HIF1alpha-PPARgamma axis Underlies the Integration of Glycolytic and Lipid Anabolic Pathways in Pathologic Cardiac Hypertrophy. Cell. Metab. 9 (6), 512–524. 10.1016/j.cmet.2009.05.005 19490906

[B21] Lage K. Greenway S. C. Rosenfeld J. A. Wakimoto H. Gorham J. M. Segrè A. V. (2012). Genetic and Environmental Risk Factors in Congenital Heart Disease Functionally Converge in Protein Networks Driving Heart Development. Proc. Natl. Acad. Sci. U. S. A. 109 (35), 14035–14040. 10.1073/pnas.1210730109 22904188 PMC3435181

[B22] Lee J. W. Ko J. Ju C. Eltzschig H. K. (2019). Hypoxia Signaling in Human Diseases and Therapeutic Targets. Exp. Mol. Med. 51 (6), 1–13. 10.1038/s12276-019-0235-1 PMC6586801 31221962

[B23] Lefterova M. I. Haakonsson A. K. Lazar M. A. Mandrup S. (2014). PPARγ and the Global Map of Adipogenesis and beyond. Trends Endocrinol. Metab. 25 (6), 293–302. 10.1016/j.tem.2014.04.001 24793638 PMC4104504

[B24] Li G. Yang L. Feng L. Yang J. Li Y. An J. (2020a). Syringaresinol Protects against Type 1 Diabetic Cardiomyopathy by Alleviating Inflammation Responses, Cardiac Fibrosis, and Oxidative Stress. Mol. Nutr. Food Res. 64 (18), e2000231. 10.1002/mnfr.202000231 32729956

[B25] Li J. Minćzuk K. Massey J. C. Howell N. L. Roy R. J. Paul S. (2020b). Metformin Improves Cardiac Metabolism and Function, and Prevents Left Ventricular Hypertrophy in Spontaneously Hypertensive Rats. J. Am. Heart Assoc. 9 (7), e015154. 10.1161/jaha.119.015154 32248762 PMC7428616

[B26] Liao W. Nguyen M. T. Yoshizaki T. Favelyukis S. Patsouris D. Imamura T. (2007). Suppression of PPAR-Gamma Attenuates Insulin-Stimulated Glucose Uptake by Affecting Both GLUT1 and GLUT4 in 3T3-L1 Adipocytes. Am. J. Physiol. Endocrinol. Metab. 293 (1), E219–E227. 10.1152/ajpendo.00695.2006 17389706

[B27] Lopaschuk G. D. Ussher J. R. (2016). Evolving Concepts of Myocardial Energy Metabolism: More Than Just Fats and Carbohydrates. Circ. Res. 119 (11), 1173–1176. 10.1161/circresaha.116.310078 28051784

[B28] Lv Z. Guo Y. (2020). Metformin and its Benefits for Various Diseases. Front. Endocrinol. (Lausanne) 11, 191. 10.3389/fendo.2020.00191 32425881 PMC7212476

[B29] MacDonald S. T. Bamforth S. D. Bragança J. Chen C. M. Broadbent C. Schneider J. E. (2013). A Cell-Autonomous Role of Cited2 in Controlling Myocardial and Coronary Vascular Development. Eur. Heart J. 34 (32), 2557–2565. 10.1093/eurheartj/ehs056 22504313 PMC3748368

[B30] Malakar A. K. Choudhury D. Halder B. Paul P. Uddin A. Chakraborty S. (2019). A Review on Coronary Artery Disease, its Risk Factors, and Therapeutics. J. Cell. Physiol. 234 (10), 16812–16823. 10.1002/jcp.28350 30790284

[B31] Marneros A. G. (2018). Effects of Chronically Increased VEGF-A on the Aging Heart. Faseb J. 32 (3), 1550–1565. 10.1096/fj.201700761RR 29146733

[B32] McCullough P. A. (2007). Coronary Artery Disease. Clin. J. Am. Soc. Nephrol. 2 (3), 611–616. 10.2215/cjn.03871106 17699471

[B33] Mohan M. Dihoum A. Mordi I. R. Choy A. M. Rena G. Lang C. C. (2021). Left Ventricular Hypertrophy in Diabetic Cardiomyopathy: A Target for Intervention. Front. Cardiovasc Med. 8, 746382. 10.3389/fcvm.2021.746382 34660744 PMC8513785

[B34] Morales D. R. Morris A. D. (2015). Metformin in Cancer Treatment and Prevention. Annu. Rev. Med. 66, 17–29. 10.1146/annurev-med-062613-093128 25386929

[B35] Nakamura M. Sadoshima J. (2018). Mechanisms of Physiological and Pathological Cardiac Hypertrophy. Nat. Rev. Cardiol. 15 (7), 387–407. 10.1038/s41569-018-0007-y 29674714

[B36] O'Rourke J. F. Tian Y. M. Ratcliffe P. J. Pugh C. W. (1999). Oxygen-regulated and Transactivating Domains in Endothelial PAS Protein 1: Comparison with Hypoxia-Inducible Factor-1alpha. J. Biol. Chem. 274 (4), 2060–2071. 10.1074/jbc.274.4.2060 9890965

[B37] Oduro P. K. Zheng X. Wei J. Yang Y. Wang Y. Zhang H. (2022). The cGAS-STING Signaling in Cardiovascular and Metabolic Diseases: Future Novel Target Option for Pharmacotherapy. Acta Pharm. Sin. B 12 (1), 50–75. 10.1016/j.apsb.2021.05.011 35127372 PMC8799861

[B38] Passariello C. L. Li J. Dodge-Kafka K. Kapiloff M. S. (2015). mAKAP-A Master Scaffold for Cardiac Remodeling. J. Cardiovasc Pharmacol. 65 (3), 218–225. 10.1097/fjc.0000000000000206 25551320 PMC4355281

[B39] Peterzan M. A. Lygate C. A. Neubauer S. Rider O. J. (2017). Metabolic Remodeling in Hypertrophied and Failing Myocardium: a Review. Am. J. Physiol. Heart Circ. Physiol. 313 (3), H597–h616. 10.1152/ajpheart.00731.2016 28646030

[B40] Pharaon L. F. El-Orabi N. F. Kunhi M. Al Yacoub N. Awad S. M. Poizat C. (2017). Rosiglitazone Promotes Cardiac Hypertrophy and Alters Chromatin Remodeling in Isolated Cardiomyocytes. Toxicol. Lett. 280, 151–158. 10.1016/j.toxlet.2017.08.011 28822817

[B41] Podhorecka M. Ibanez B. Dmoszyńska A. (2017). Metformin - its Potential Anti-cancer and Anti-aging Effects. Postepy Hig. Med. Dosw (Online) 71 (0), 170–175. 10.5604/01.3001.0010.3801 28258677

[B42] Rymer J. A. Rao S. V. (2018). Anemia and Coronary Artery Disease: Pathophysiology, Prognosis, and Treatment. Coron. Artery Dis. 29 (2), 161–167. 10.1097/mca.0000000000000598 29280914

[B43] Sanchez-Rangel E. Inzucchi S. E. (2017). Metformin: Clinical Use in Type 2 Diabetes. Diabetologia 60 (9), 1586–1593. 10.1007/s00125-017-4336-x 28770321

[B44] Semenza G. L. (2014). Hypoxia-inducible Factor 1 and Cardiovascular Disease. Annu. Rev. Physiol. 76, 39–56. 10.1146/annurev-physiol-021113-170322 23988176 PMC4696033

[B45] Sorop O. van de Wouw J. Chandler S. Ohanyan V. Tune J. D. Chilian W. M. (2020). Experimental Animal Models of Coronary Microvascular Dysfunction. Cardiovasc Res. 116 (4), 756–770. 10.1093/cvr/cvaa002 31926020 PMC7061277

[B46] Sousa Fialho M. D. L. Abd Jamil A. H. Stannard G. A. Heather L. C. (2019). Hypoxia-inducible Factor 1 Signalling, Metabolism and its Therapeutic Potential in Cardiovascular Disease. Biochim. Biophys. Acta Mol. Basis Dis. 1865 (4), 831–843. 10.1016/j.bbadis.2018.09.024 30266651

[B47] Tang X. Chen X. F. Wang N. Y. Wang X. M. Liang S. T. Zheng W. (2017). SIRT2 Acts as a Cardioprotective Deacetylase in Pathological Cardiac Hypertrophy. Circulation 136 (21), 2051–2067. 10.1161/circulationaha.117.028728 28947430 PMC5698109

[B49] Tian W. Yang L. Liu Y. He J. Yang L. Zhang Q. (2020). Resveratrol Attenuates Doxorubicin-Induced Cardiotoxicity in Rats by Up-Regulation of Vascular Endothelial Growth Factor B. J. Nutr. Biochem. 79, 108132. 10.1016/j.jnutbio.2019.01.018 30857673

[B50] Tirziu D. Chorianopoulos E. Moodie K. L. Palac R. T. Zhuang Z. W. Tjwa M. (2007). Myocardial Hypertrophy in the Absence of External Stimuli Is Induced by Angiogenesis in Mice. J. Clin. Invest. 117 (11), 3188–3197. 10.1172/jci32024 17975666 PMC2045601

[B51] Wali J. A. Jarzebska N. Raubenheimer D. Simpson S. J. Rodionov R. N. O'Sullivan J. F. (2020). Cardio-Metabolic Effects of High-Fat Diets and Their Underlying Mechanisms-A Narrative Review. Nutrients 12 (5), 1505. 10.3390/nu12051505 PMC7284903 32455838

[B52] Wang G. L. Jiang B. H. Rue E. A. Semenza G. L. (1995). Hypoxia-inducible Factor 1 Is a Basic-Helix-Loop-Helix-PAS Heterodimer Regulated by Cellular O2 Tension. Proc. Natl. Acad. Sci. U. S. A. 92 (12), 5510–5514. 10.1073/pnas.92.12.5510 7539918 PMC41725

[B53] Wang X. Yang L. Kang L. Li J. Yang L. Zhang J. (2017). Metformin Attenuates Myocardial Ischemia-Reperfusion Injury *via* Up-Regulation of Antioxidant Enzymes. PLoS One 12 (8), e0182777. 10.1371/journal.pone.0182777 28817623 PMC5560646

[B54] Wen Y. Zhou X. Lu M. He M. Tian Y. Liu L. (2019). Bclaf1 Promotes Angiogenesis by Regulating HIF-1α Transcription in Hepatocellular Carcinoma. Oncogene 38 (11), 1845–1859. 10.1038/s41388-018-0552-1 30367150 PMC6462866

[B55] Yamamoto T. Endo J. Kataoka M. Matsuhashi T. Katsumata Y. Shirakawa K. (2020). Palmitate Induces Cardiomyocyte Death *via* Inositol Requiring Enzyme-1 (IRE1)-Mediated Signaling Independent of X-Box Binding Protein 1 (XBP1). Biochem. Biophys. Res. Commun. 526 (1), 122–127. 10.1016/j.bbrc.2020.03.027 32199617

[B56] Yan X. L. Wang Y. Y. Yu Z. F. Tian M. M. Li H. (2018). Peroxisome Proliferator-Activated Receptor-Gamma Activation Attenuates Diabetic Cardiomyopathy *via* Regulation of the TGF-β/ERK Pathway and Epithelial-To-Mesenchymal Transition. Life Sci. 213, 269–278. 10.1016/j.lfs.2018.09.004 30189217

[B57] Yerevanian A. Soukas A. A. (2019). Metformin: Mechanisms in Human Obesity and Weight Loss. Curr. Obes. Rep. 8 (2), 156–164. 10.1007/s13679-019-00335-3 30874963 PMC6520185

[B58] Zhang H. Cai L. (2020). Zinc Homeostasis Plays an Important Role in the Prevention of Obesity-Induced Cardiac Inflammation, Remodeling and Dysfunction. J. Trace Elem. Med. Biol. 62, 126615. 10.1016/j.jtemb.2020.126615 32683230

[B59] Zhang Y. Liu X. Zhang L. Li X. Zhou Z. Jiao L. (2018). Metformin Protects against H2O2-Induced Cardiomyocyte Injury by Inhibiting the miR-1a-3p/GRP94 Pathway. Mol. Ther. Nucleic Acids 13, 189–197. 10.1016/j.omtn.2018.09.001 30292140 PMC6172474

[B60] Zhou J. Zhang W. Liang B. Casimiro M. C. Whitaker-Menezes D. Wang M. (2009). PPARgamma Activation Induces Autophagy in Breast Cancer Cells. Int. J. Biochem. Cell. Biol. 41 (11), 2334–2342. 10.1016/j.biocel.2009.06.007 19563910 PMC2753745

[B61] Zhu M. Zhang Q. Wang X. Kang L. Yang Y. Liu Y. (2016). Metformin Potentiates Anti-tumor Effect of Resveratrol on Pancreatic Cancer by Down-Regulation of VEGF-B Signaling Pathway. Oncotarget 7 (51), 84190–84200. 10.18632/oncotarget.12391 27705937 PMC5356654

[B62] Zilov A. V. Abdelaziz S. I. AlShammary A. Al Zahrani A. Amir A. Assaad Khalil S. H. (2019). Mechanisms of Action of Metformin with Special Reference to Cardiovascular Protection. Diabetes Metab. Res. Rev. 35 (7), e3173. 10.1002/dmrr.3173 31021474 PMC6851752

